# Time has come to include Human Papillomavirus (HPV) testing in sperm donor banks

**Published:** 2018-12

**Authors:** CE Depuydt*, GGG Donders, L Verstraete, D Vanden Broeck, JFA Beert, G Salembier, E Bosmans, N DhontT, I Van Der Auwera, K Vandenborne, W Ombelet

**Affiliations:** Department of Hormonology and Reproductive Health, AML, Sonic Healthcare, Antwerp, Belgium;; Intermediate structure for human body material, AML, Sonic Healthcare, Antwerp, Belgium;; Femicare, Clinical Research for Women, Tienen, Belgium;; Department of Obstetrics and Gynecology, Regional Hospital Heilig Hart, Tienen, Belgium;; University Hospital Antwerpen, Antwerp, Belgium;; Department of Clinical and Molecular Pathology, AML, Sonic Healthcare, Antwerp, Belgium; 7; National Reference Centre for HPV, Brussels, Belgium;; Department of Obstetrics and Gynecology, International Centre for Reproductive Health, Ghent University, Ghent;; Genk Institute for Fertility Technology, ZOL Hospitals, Genk, Belgium;; UHasselt, Faculty of Medicine and Life Sciences, LCRC, Diepenbeek, Belgium.

**Keywords:** infertility, infectious, intrauterine insemination, transient virion producing, spermatozoa, semen, donor sperm

## Abstract

HPV is well known as a potential cause of cervical cancer. Less well known is its link to temporal subfertility that is caused by binding of infectious virions to the spermatozoa’s head which induces sperm-DNA damage and causes a reduction in clinical pregnancy rates in women receiving HPV positive semen.

This impact on the global fertility burden remains greatly underestimated and underexplored. This risk of reduced fertility due to infectious HPV in sperm is especially important when donor sperm insemination is considered, since testing for the presence of HPV virions before use seems warranted.

We tested 514 donor sperm samples from 3 different sperm banks for 18 different HPV types.

Overall 3.9% (20/514) of tested donor sperm was positive for HPV, with different prevalence among the 3 different sperm banks (3.6% bank A, 3.1% bank B and 16.7% bank C). Also the HPV virion per spermatozoon ratio in donor samples was similar across the different sperm banks (95% CI 0,01 to 1,07 HPV virions/spermatozoon).

When HPV positive donor sperm was used, no clinical pregnancies resulted, whereas when HPV negative donor sperm was used the clinical pregnancy rate was 14.6%.

From both a cost/benefit and a safety point of view we recommend that donor sperm should always be tested for HPV before using it for insemination.

## Introduction

Although infection with human papillomavirus (HPV) is very common worldwide, until recently there was only scarce evidence linking HPV infection to reduced fertility outcomes (Garolla et al., 2016; Gizzo et al., 2014; Xiong et al., 2018).

One of the major reasons why it took so long to realize infectious HPV virions impair fertility is because formerly we often failed to see the different impact of infection and disease, HPV viruses and HPV virions (Depuydt et al., 2016a). On one hand HPV can cause the well-known but rather uncommon transformation of HPV infected dividing cells into cancer on the uterine cervix or other organs. These HPV morphotypes are incorporated into the cell’s DNA and are no longer infectious ( Depuydt et al., 2012b; 2015; 2016b). On the other hand, the more frequent free infectious HPV virions can bind the spermatozoa’s head (Foresta et al., 2011b; Kaspersen et al., 2011), induce sperm DNA damage (Boeri et al., 2019), causing temporal subfertility demonstrated by reduced clinical pregnancy rates in subfertile women receiving inseminations with HPV positive semen (Depuydt et al., 2019). It is often overlooked that the bulk of detected HPV DNA is infectious in origin (Depuydt et al., 2016a) and its impact on the global fertility burden is greatly underestimated as evidenced by a recent study that showed that the presence of HPV virions in semen significantly decrease clinical pregnancy rates in women undergoing intrauterine insemination (Depuydt et al., 2019).

Because of the absence of dividing cells in semen, the measured HPV DNA in sperm samples always originates from virions that can only be produced in non-dividing desquamating cells. Despite the fact that only 1-10 virions are sufficient to infect a basal cell and establish infection (Patterson et al., 2005), only a few authors have tested HPV prevalence in donor sperm (Kaspersen et al., 2011; D’Hauwers et al., 2012) or suggested screening of donor semen for HPV to prevent iatrogenic cervical HPV infections in the recipient (Basky G, 2000; Foresta et al., 2011a).

It has been reported before that HPV virions can physically bind to the spermatozoon’s head (Foresta et al., 2011b; Kaspersen et al., 2011) and induce sperm DNA damage (Boeri et al., 2019). In their latest guidelines, the Belgian superior health council does not recommend routine HPV testing of donor sperm, because at that time it remained unclear what the exact implications of HPV positive spermatozoa were for cell biology (Superior Health Council, 2014), indicating an update could be recommended. Although the sperm donors are selected to have good quality sperm (having children) and are labeled healthy, still a percentage can be positive for oncogenic HPV types or other HPV types that impact fertilization successes. In the past, no correlation was made on how often or how frequently HPV positive donor sperm was successful in achieving pregnancies, as compared to the HPV negative donor sperm (Kaspersen et al., 2013). We recently showed in a blinded non-interventional prospective study that women who were inseminated with HPV positive sperm had reduced clinical pregnancy rates compared to women who received HPV negative sperm.

In order to formulate a recommendation regarding HPV donor sperm testing for sperm banks, we assessed the impact of the HPV status on success rates of donor sperm by analyzing donor sperm that was used for inseminations originating from 3 different donor banks.

## Methods and Materials

### Study population

We measured HPV DNA in donor sperm that was destined for IUI as previously described (Depuydt et al., 2019). Briefly, depending on the capacitation procedure used (washing away the seminal plasma with capacitation medium, swim-up or density gradient), different left-over sperm fractions were obtained from donor sperm. Work-up of the 514 donor sperm samples generated 662 separate sperm fractions (151 sperm fractions after wash or density gradient and seminal plasma from 511 samples).

If in one of the sperm fractions HPV DNA was detected, the sperm sample was considered HPV positive. Donor sperm originated from one of three donor banks (A: n=365, B: n=131 and C: n=18). The study was approved by both the Institutional Review Board of the University hospital of Antwerp and the University of Antwerp (Belgian registration number: B300201733597; Eudra CT number: 2017-004791-56).

### Real-time type-specific quantitative PCR (qPCR) analysis of HPV DNA in donor sperm

DNA extraction on donor sperm was performed on the ABBOTT M200sp as previously described (Depuydt et al., 2019). Each DNA extract was subjected to a clinically validated real-time quantitative PCR assay (Depuydt et al., 2012a) for the detection of 18 different HPV types: HPV6 E6, HPV11 E6, HPV16 E7, HPV18 E7, HPV31 E6, HPV33 E6, HPV35 E6, HPV39 E7, HPV45 E7, HPV51 E7, HPV52 E7, HPV53 E6, HPV56 E7, HPV58 E7, HPV59 E7, HPV66 E6, HPV67 L1 and HPV68 E7 as previously described by Micalessi et al. (2012). HPV prevalence was defined as the presence of one or more of the above mentioned HPV types. High-risk HPV infection (HR HPV) was defined as the presence of one or more of the following HPV types: HPV16, HPV18, HPV31, HPV33 HPV35, HPV39, HPV45, HPV51, HPV52, HPV56, HPV58, HPV59, and HPV68 (Munoz et al., 2003), possible HR (pHR) HPV when one of the following HPV types was present: HPV type 53 and HPV type 66. Low-risk HPV infection (LR HPV) was defined as the presence of one or more of the following HPV types: HPV6, HPV11 and HPV67. A ß-globin real-time quantitative PCR was used to assess the DNA quality and to estimate the number of cells (Depuydt et al., 2006). The analytical sensitivity of the different type specific HPV qPCRs varies between 1 and 100 HPV copies/qPCR reaction (Depuydt et al., 2007; 2012a).

### The HPV virion per spermatozoon ratio

The number of HPV copies (virions) per ml sperm was divided by the number of spermatozoa per ml sperm to calculate the virions to spermatozoon ratio (HPV copies/spermatozoon). When different HPV types were present in one sperm sample, the number of HPV copies per ml of sperm for each individual HPV type was added and divided by the number of spermatozoa per ml as previously described (Depuydt. et al., 2019).

### Statistical analysis

Statistical analysis was performed with MedCalc® (MedCalc Software, Ostend, Belgium) (Schoonjans et al., 1995).

## Results

### HPV prevalence in donor sperm from 3 different sperm banks

From the 365 donor sperm samples tested from bank A, 13 tested positive for one or two HPV types (3.6%). From bank B, 4 donor sperm samples tested HPV positive out of 131 (3.1%) and from bank C, 3 samples tested positive out of 18 (16.7%; Table I).

**Table I:** HPV positivity in donor sperm from 3 different sperm banks.

Sperm bank	HPV in donor sperm	# straws	%
A	Negative	352	96.4
	Positive	13	3.6
	Total	365	100
			
B	Negative	127	96.9
	Positive	4	3.1
	Total	131	100
			
C	Negative	15	83.3
	Positive	3	16.7
	Total	18	100
			
All	Negative	494	96.1
	Positive	20	3.9
	Total	514	100

HPV negative = negative for following HPV types: HPV 6, 11, 16, 18, 31, 33, 35, 39, 45, 51, 52, 53, 56, 58, 59, 66, 67 and 68. HPV positive = positive for one of following HPV types: HPV 16, 18, 31, 39, 51, 52, 58, 66 and 67.

### HPV prevalence in donor sperm

From the 514 donor sperm samples tested, 20 (3.9%) tested positive for at least one HPV type and 2 (0.4%) tested positive for two HPV types (Table II). The majority of the detected HPV types were HR (high risk) HPV types, with HPV 31 as the most frequently detected HR HPV type (5/20; 25%). From the pHR and LR (low risk) HPV types only HPV types 66 and 67 were detected.

**Table II:** Prevalence of different HPV types detected in donor sperm.

HPV type	n	%
6	ND	0
11	ND	0
16	ND	0
16.51	1	5.0
18	2	10.0
18.67	1	5.0
31	5	25.0
33	ND	0
35	ND	0
39	2	10.0
45	ND	0
51	ND	0
52	1	5.0
53	ND	0
56	ND	0
58	3	15.0
59	ND	0
66	1	5.0
67	4	20.0
68	ND	0
Total	20	100.0

LR HPV: low risk HPV types 6, 11 and 67; pHR HPV: possible high risk HPV types 53 and 66; HR HPV: high risk HPV types 16, 18, 31, 33, 35, 39, 45, 51, 52, 56, 58, 59 and 68; ND = not detected.

### The HPV virion per spermatozoon ratio

**Figure 1:**
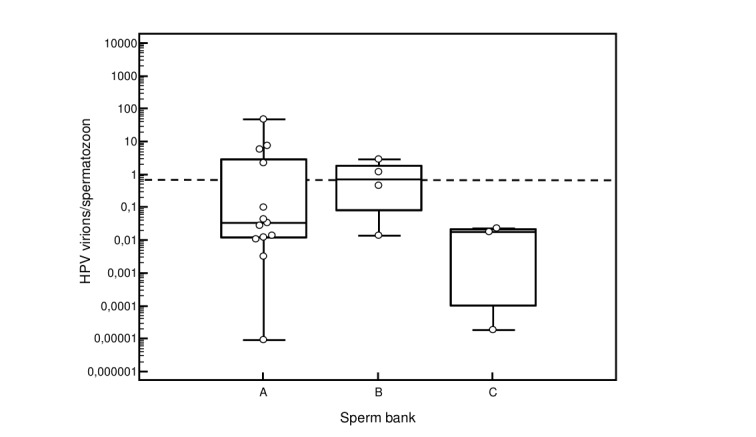
HPV virion per spermatozoon ratio in HPV positive donor sperm according to sperm bank. The dashed line represents the 0,66 HPV virions per spermatozoon cut-off above which no clinical pregnancies could be achieved when HPV positive sperm was used for IUI (ref Depuydt Fertil Steril). Following HPV types were quantified with real time qPCR: HPV 6,11,16,18,31,33,35,39,45,51,52,53,56,58,59,67 and 68. When different HPV types were present in donor sperm, the number of copies per ml of sperm for each individual HPV type was added and divided by the number of spermatozoa per ml.

The median HPV virion per spermatozoon ratio in HPV positive donor sperm was 0.03 (95% CI 0.01 to 1.07 HPV virions/spermatozoon). There was no difference between the median HPV virion per spermatozoon ratio of HPV positive donor sperm from the 3 different sperm banks (Figure 1). We previously showed that pregnancies can still occur for both homologous and donor sperm inseminations when the virion per spermatozoon ratio is below the threshold of 0,66 HPV virions per spermatozöon (Depuydt et al., 2019; Figure 1).

Although most donor samples (14/20) had a HPV virion per spermatozoon ratio below this cut-off ratio of 0.66 HPV virions per spermatozoon, still no clinical pregnancies were achieved with HPV positive donor sperm. Whereas insemination with HPV negative donor sperm led to clinical pregnancies in 14.6% per IUI cycle.

## Discussion

Our data on HPV typing of 514 semen samples from sperm donors originating from 3 different sperm banks clearly shows that HPV positive donor sperm was found in all three sperm banks. In total 20 donor sperm samples tested positive for HPV (3.9%), comparable to the 4.8% HPV prevalence previously found in the sperm bank of the University Hospital of Antwerp (D’Hauwers et al., 2012). The variation of HPV positivity of donor sperm within each of the tested sperm banks might be explained by the heterogeneity in sperm banking facilities in Belgium (Thijssen et al., 2014). Additionally, large commercial sperm banks have a greater donor pool than smaller local sperm banks and can therefore afford to select only the very best donors. This results in better sperm quality from large sperm banks which is probably linked to lower HPV sperm prevalence.

None of the inseminations from the HPV positive donors in the current study resulted in a pregnancy (Depuydt et al., 2019), as compared to 14.6% pregnancies resulting from inseminations with HPV negative donor sperm.

According to the results report generated from European registers by ESHRE for the year 2012 (Calhaz-Jorge et al., 2016) a total of 43.497 IUI with donor semen have been performed. Extrapolating from our observed frequencies, this would lead to around 1696 HPV infected donor semen samples used in IUI per year in Europe. This is however an underestimation since many cycles are not registered in the European registers because the insemination is done privately, and donor sperm can be bought by patients directly from international sperm banks due to the free movement of persons and goods in Europe.

In our opinion, donor sperm should be systematically screened for HPV, not only from a cost/benefit point of view, but also for safety reasons. Indeed since no dividing cells are present in sperm, HPV DNA detected in sperm always originates from infectious HPV virions, potentially resulting in transmission and potential infection of the inseminated women. As a consequence, most women (81%) (Depuydt et al., 2016a) will develop transient HPV virion production, prolonging their temporal subfertility, while one fifth (19%) of women will develop a clonal HPV transformed HPV infection, 1/3 of which can lead to cervix cancer (Verhelst et al., 2016). Therefore, although most induced HPV infections in those women who were infected with HPV positive donor sperm are allegedly transient or will regress in time, still a small fraction however is at risk to develop invasive cervical cancer due to insemination with HPV positive donor sperm. Because the HPV virions can bind the spermatozoa (Foresta et al., 2011b; Kaspersen et al., 2011) and efforts to use sperm preparation techniques to remove the virions were not successful (Brossfield et al., 1999; Chan et al., 1994; Olatunbosun et al., 2001), excluding sperm donations with HPV positive sperm from the banks could prevent transmission of HPV infections.

Based on these data we strongly recommend from both a cost/benefit and a safety point of view that donor sperm should always be tested for HPV prior to insemination.
